# Schema Compliant Consistency Management via Triple Graph Grammars and Integer Linear Programming

**DOI:** 10.1007/978-3-030-45234-6_16

**Published:** 2020-03-13

**Authors:** Nils Weidmann, Anthony Anjorin

**Affiliations:** 8 University of Paderborn, Paderborn, Germany; 9 ICREA, Open University of Catalonia, Barcelona, Spain; Paderborn University, Paderborn, Germany

**Keywords:** Application conditions, Triple graph grammars, Integer linear programming

## Abstract

Triple Graph Grammars (TGGs) are a declarative and rule-based approach to bidirectional model transformation. The key feature of TGGs is the automatic derivation of various operations such as unidirectional transformation, model synchronisation, and consistency checking. Application conditions can be used to increase the expressiveness of TGGs by guaranteeing schema compliance, i.e., that domain constraints are respected by the TGG. In recent years, a series of new TGG-based operations has been introduced leveraging Integer Linear Programming (ILP) solvers to flexible consistency maintenance even in cases where no strict solution exists. Schema compliance is not guaranteed, however, as application conditions from the original TGG cannot be directly transferred to these ILP-based operations. In this paper, we extend ILP-based TGG operations so as to guarantee schema compliance. We implement and evaluate the practical feasibility of our approach.

## 1 Introduction

In the context of Model-Driven Engineering (MDE), software systems are represented as a collection of different models. Often several semantically related models are involved and therefore have to be kept consistent to each other. The process of maintaining consistency among multiple models is called consistency management and involves various operations including (unidirectional) transformation, synchronisation, and consistency checking. Practical applications of consistency checking occur in the industry automation domain, where multiple domain-specific languages (DSLs) are used to describe complex systems [4].

Triple Graph Grammars (TGGs) are a declarative rule-based approach to specifying a bidirectional consistency relation between two modelling languages. The main advantage of TGGs is the possibility to derive multiple consistency management operations from the same formal specification. In their roadmap for

* This work was partially supported by the German Federal Ministry of Education and Research (BMBF) through the SPEAR project (01IS17024I).

future research on TGGs [2], Anjorin et al. name the *expressiveness* of the TGG language in use as one research dimension. One way of increasing the expressiveness [25] of TGGs is to ensure the satisfaction of certain *constraints*, such as multiplicities with lower and upper bounds, which are typically posed by each domain and should be respected by consistency maintainers. Using terminology from Ehrig et. al [9], so called graph constraints consist of a premise (if), and a set of conclusions (then). They are powerful enough to *forbid* certain situations (negative constraints), *demand* certain conditions (positive constraints), and *enforce* implications. One possible approach to handling constraints in the context of TGGs is the use of *application conditions (ACs)* to restrict the applicability of rules. The subset of ACs supported for operationalised TGGs is, however, still quite restricted. All approaches we are aware of only handle a subset of Negative Application Conditions (NACs) and mostly focus on model transformation and synchronisation rather than consistency checking.

Recent work [17,18,20,24] has introduced TGG operations based on Integer Linear Programming (ILP). Such operations are advantageous because they implement a flexible and generic strategy for multiple consistency management operations, while still providing acceptable scalability for growing model sizes. Flexibility here means that the consistency management operations are able to handle cases where no strict solution exists by providing "optimal" partial results. Graph constraints, however, have not yet been integrated in this hybrid ILP-TGG framework and only basic TGG language features [25] are currently supported. We extend this line of work by the notion of *schema compliance* for TGGs, i.e., that all derived operations respect a set of constraints, as introduced by Anjorin et al. [3]. Instead of trying to integrate ACs into TGG rules, we propose to handle domain constraints directly in the ILP-based operations, thus achieving schema compliance in this manner. By directly encoding graph constraints as ILP constraints, we are able to handle a larger class of constraints than in previous work on schema compliance [3]. We apply our approach to consistency checking with given correspondence links: a basic operation that must be both flexible and efficient as it is often used as a "cheap" check in order to avoid unnecessary work and ensure hippocraticness [6]. An extension to other operations such as unidirectional transformation is straightforward and sketched at the end of this paper. Our approach can be regarded as a step towards tolerant consistency management, as the largest consistent sub-triple is computed in case of inconsistent input models. In this case, checking all domain constraints in advance is not helpful as the user is only informed about the violation of constraints and is not provided with a partial but optimal result.

The rest of the paper is organised as follows: Section 2 introduces a running example, which is used to explain the main ideas on an intuitive level in Sect. 3. Our contribution is compared with related work in Sect. 4. Basic definitions are provided in Sect. 5, and used to express the formal concepts in Sect. 6. A reference implementation together with an experimental evaluation is described in Sect. 7, before discussing extensions towards other operations in Sect. 8. Finally, Sect. 9 concludes the paper and provides some directions for future work.

## 2 Running Example

To illustrate our approach, a consistency relation between simplified data models of the social networks *Facebook* and *Instagram* is used as a running example. The respective metamodels are depicted in Fig. 1. A `FacebookNetwork` consists of multiple `FacebookUsers`, who can share `Friendships` with each other. Similarly, an `InstagramNetwork` is made up of arbitrarily many `InstagramUsers`. In contrast to the `Facebook` metamodel, the social interaction is not expressed via `Friendship` nodes but by a `follows` relation between `InstagramUsers`. To complete the triple, a correspondence metamodel connects the network and user classes of the two metamodels via correspondence types, depicted as hexagons. In the following diagrams, the prefixes `Facebook` and `Instagram` are abbreviated with `F` and `I`, respectively. A triple graph typed according to Fig. 1 is consistent if (1) the correspondence links form a bijection between all networks and users of the two networks, and (2) the following additional graph constraints are satisfied:

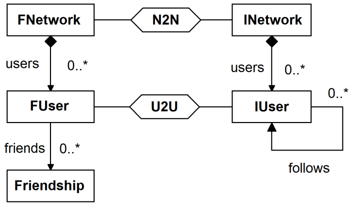

**Fig. 1.** Triple of Metamodels

- We *forbid* two or more `Friendship` nodes connecting the same two `FacebookUsers` as depicted in Fig. 2. This is denoted as a *negative constraint.*
- There should be a `Friendship` between two `FacebookUsers` if the corresponding `InstagramUsers` follow each other. This means if the *premise* that two `InstagramUsers` follow each other holds, the *conclusion* that there is a corresponding `Friendship` on `Facebook` should also hold. The combination of premise and (possibly multiple) conclusions is denoted as *positive constraint* (as depicted in Fig. 3).

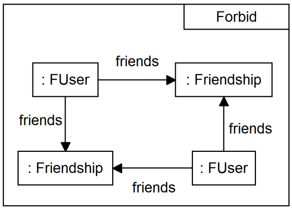

**Fig. 2.** `NoDoubleFriendship`

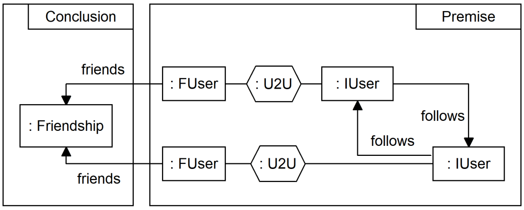

**Fig. 3.** `EnforceFriendship`

## 3 Main Ideas

In this section, we demonstrate our approach by formalising the consistency relation from the running example as a TGG and deriving a consistency checker. The novelty of our approach is that we are able to guarantee schema compliance, i.e., that all additional graph constraints (two from the running example) are respected by the consistency checker.

The consistency relation can be defined by four TGG rules depicted in Fig. 4, 5, 6, and 7. Nodes and edges required as context (i.e., they have to be matched to apply the rule) are depicted in black, while elements created by the rule are depicted in green and are annotated with a `++`-markup. Accordingly, the rule `NetworkToNetwork` creates a `FacebookNetwork` and a corresponding `InstagramNetwork`, whereas `UserToUser` creates corresponding users, requiring corresponding networks as context. The other two rules add relationships between two users in the two social networks. `RequestFriendship` creates a `follows` edge in the `Instagram` model, while the `Facebook` model remains unchanged. A `follows` edge in the opposite direction is added between two `InstagramUsers` and a `Friendship` node is created for the corresponding `FacebookUsers` when the rule `AcceptFriendship` is applied. A triple graph is consistent if it can be generated using the four rules of the TGG and if it fulfils the two graph constraints.

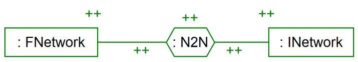

**Fig. 4.** Rule `NetworkToNetwork`

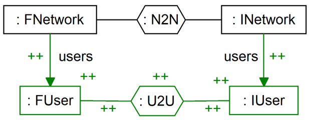

**Fig. 5.** Rule `UserToUser`

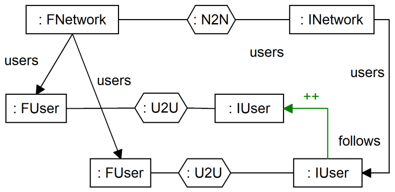

**Fig. 6.** Rule `RequestFriendship`

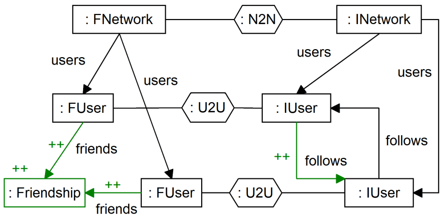

**Fig. 7.** Rule `AcceptFriendship`

To determine if a given triple is contained in the language of a TGG and fulfils all additional graph constraints, we try to find a set of rule applications that marks the input triple entirely while fulfilling all generated ILP constraints. If this is impossible, we conclude that the given triple is inconsistent and provide a consistent sub-triple with maximum number of elements as result. Five constraint types and the construction of the objective function are briefly introduced using the example instance depicted in Fig. 8 which can be generated by the TGG but violates the constraint `NoDoubleFriendship`. The elements are annotated with variables which correspond to those rules that potentially mark the respective element, i.e. `NetworkToNetwork` ($d_1$), `UserToUser` ($d_2, d_3$), `RequestFriendship` ($d_4$, $d_5$) and `AcceptFriendship` ($d_6$, $d_7$). A variable is set to 1 if the associated rule application is chosen to be applied to create the solution graph. Furthermore, Fig. 8 also depicts all matches for `NoDoubleFriendship` ($p_8$), the premise of `EnforceFriendship` ($p_9$)[1] and the conclusion for `Enforce-Friendship` ($c_{10}, c_{11}$). To allow for uniform handling, negative constraints are represented as graph constraints with a premise but no conclusions.

**Context for rules:** The applicability of rules that require elements as context depends on previous rule applications that have created these elements. In the example instance, the application of `UserToUser` ($d_2, d_3$) implies that the rule `NetworkToNetwork` ($d_1$) was applied already, because the `INetwork` is required as context. ILP implication constraints of the form $di \implies (d_{j_1} \vee \cdots \vee d_{j_m}) \wedge \cdots \wedge (d_{k_1} \vee \cdots \vee d_{k_n})$ are thus created for all rules applications $d_i$ with required context elements $j, \ldots, k$, and rule applications $(d_{j_1}, \ldots, d_{j_m}, \ldots, d_{k_1}, \ldots d_{k_n})$ that can mark these elements.

**Exclusions for rules:** As elements should only be marked once, multiple rule applications that mark the same element exclude each other. The `follows` edges between two `InstagramUsers` can be marked both by applications of `RequestFriendship` ($d_4$, $d_5$) and `AcceptFriendship` ($d_6$, $d_7$). For each element that can be marked by multiple rule applications $d_i, \ldots, d_j$, an ILP exclusion constraint $d_i \oplus \cdots \oplus d_j$ is created.

**Context for premises:** Similar to ILP implication constraints for rules, matches for the premises of graph constraints also depend on context provided by other rule applications (whereas no elements are marked by those matches, so there are no context dependencies among them). However, as soon as the context is provided completely, the premise *is* fulfilled. The implication constraint is thus in the opposite direction: Choosing a subset of rule applications $d_i, \ldots, d_j$ that is sufficient to create the context for a premise match $p_k$ implies that $p_k$ has to be chosen.

**Context for conclusions:** For a conclusion of a graph constraint to hold, all required elements have to be marked, which is reflected in a constraint similar to the context constraint for rules. In the concrete example, there are two matches ($c_{10}$, $c_{11}$) for the conclusion of `EnforceFriendship` (differing in `F1` and `F2` as `Friendship` nodes).

**Implications for graph constraints:** The semantics of premise and conclusion(s) is reflected in the implications for graph constraints, which define that the presence of a premise match implies the existence of a corresponding conclusion match. $p_8$ as a negative constraint is represented as a graph constraint with a premise but no conclusions, whereas $p9$ implies $c_{10}$ or $c_{11}$ to be satisfied.

**Objective function:** In order to find a consistent solution for the given input, it is necessary to find a set of rule applications that marks the input models *entirely*. The objective function maximizes the number of marked elements, i.e. each variable associated with a rule application is weighted with the number of elements it marks, and the weighted sum is maximised. Variables associated with constraints need not be taken into account because they do not create elements.

[1] To simplify the solution, we omit symmetric matches that lead to more ILP constraints but neither change the result nor provide additional insight.

Context for rules:

- $d_2 \implies d_1$
- $d_3 \implies d_1$
- $d_4 \implies d_1 \wedge d_2 \wedge d_3$
- $d_5 \implies d_1 \wedge d_2 \wedge d_3$
- $d_6 \implies d_1 \wedge d_2 \wedge d_3 \wedge d_5$
- $d_7 \implies d_1 \wedge d_2 \wedge d_3 \wedge d_4$

Exclusions for rules:

- $d_4 \oplus d_6$
- $d_5 \oplus d_7$

Context for premises:

- $d_2 \wedge d_3 \wedge d_6 \wedge d_7 \implies p_8$
- $d_2 \wedge d_3 \wedge (d_4 \vee d_6) \wedge (d_5 \vee d_7) \implies p_9$

Context for conclusions:

- $c_{10} \implies d_2 \wedge d_3 \wedge (d_4 \vee d_6) \wedge (d_5 \vee d_7) \wedge d_6$
- $c_{11} \implies d_2 \wedge d_3 \wedge (d_4 \vee d_6) \wedge (d_5 \vee d_7) \wedge d_7$

Implications for graph constraints:

- $p_8 \implies \text{false}$
- $p_9 \implies c_{10} \vee c_{11}$

Objective Function: max. $3d_1 + 5d_2 + 5d_3 + d_4 + d_5 + 4d_6 + 4d_7$

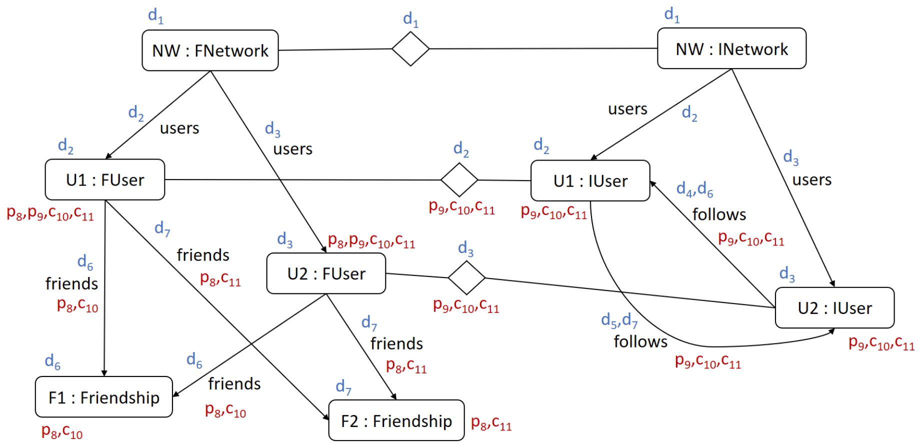

**Fig. 8.** Example instance with annotations for rule applications and constraint matches

All context elements in the example instance can be marked setting $d_1$, $d_2$, $d_3$, $d_6$ and $d_7$ to 1 and $d_4$ and $d_5$ to 0, leading to an objective function value of 21 equal to the total number of elements. This marking would however violate the constraint `NoDoubleFriendship`, as `U1` and `U2` are connected by two `Friendship` nodes. This violation is reflected in the ILP constraints as well: The first context constraint for premises enforces setting $p_8$ to 1, which immediately contradicts the first implication for graph constraints. As no other subset of rule applications is able to mark the input triple entirely, the consistency check fails. The optimal solution, representing the maximal consistent sub-triple, is achieved either by exchanging $d_4$ and $d_6$ or $d_5$ and $d_7$ in the set of chosen rule applications, decreasing the objective function value to 18 and leaving one `Friendship` node and the two connecting `friends` edges unmarked. Note that for this example, the objective function and hard constraints contradict each other, emphasising the fact that constraints must be taken into account when computing optimal partial solutions.

## 4 Related Work

Our contribution builds upon and extends the existing work on combining TGGs and ILP [17, 18, 20, 24]. This previous work covers the basic idea of modelling consistency checking without correspondence links as a search problem [17, 20], a proof for correctness and completeness [18], and a generalisation to include other operations such as unidirectional transformation and consistency checking with correspondence links [24]. Only basic TGG rules without graph constraints or ACs are handled, meaning that schema compliance cannot be guaranteed.

To the best of our knowledge, all existing TGG-based approaches ensure schema compliance by enriching a provided TGG with suitable ACs. Ehrig et al. introduce NACs to TGG and prove correctness and completeness for unidirectional model transformation [10]. Golas et al. [13] extend these results to more general ACs for TGGs but only cover the direct application of TGG rules, i.e., model triple generation. In both cases, the runtime efficiency and thus practical feasibility of the derived operations is beyond scope. With a focus on guaranteeing polynomial runtime, Klar et al. [16] present a translation algorithm with polynomial runtime for correct and complete TGG-based unidirectional model transformation. Klar et al. restrict the class of supported NACs to NACs that are only used to guarantee schema compliance, arguing that (i) such NACs can be supported efficiently, (ii) are still very useful in practice to guarantee schema compliance, and (iii) can also be efficiently supported by model synchronisation algorithms (as later demonstrated [19]). Anjorin et al. [3] show that this restricted class of "schema compliance" NACs can be automatically generated from negative constraints and is thus equivalent to providing negative constraints together with a TGG. All these approaches, however, can only handle negative constraints that are contained in a single domain, as the derivation of forward and backward transformations can only handle "domain separable" NACs.

Similar to our hybrid TGG/ILP-approch, Callow and Kalawski [5] combine model transformation and Mixed Integer Linear Programming (MILP) optimization techniques but focus on model compliance for forward transformations and not on deriving multiple consistency management operations. Xiong et al. [26] solve consistency management tasks using the Haskell-based language Beanbag. The approach considers implicit constraints and correspondences and is tailored to the application to Unified Modeling Language (UML) structures, though.

There are also purely constraint-based approaches [11, 14, 21] that encode both model structure and consistency relation into constraints and can easily handle schema compliance. This comes at a price, however, as the underlying constraint solvers do not scale with model-size and cannot compete with other approaches [1]. Our hybrid TGG/ILP approach is a compromise that leverages the flexibility of constraint solvers but still scales reasonably well [24] as the variables of the ILP problem are matches and not model elements.

There are also various constraint-based approaches that use bio-inspired meta-heuristics and could also handle schema compliance. The tool MOMoT [12] realises model transformation based on evolutionary algorithms as a search strategy for rule orchestration. Similarly, the multi-objective optimisation technique

Design Space Exploration (DSE) is used by Denil et al. [7] in combination with the T-core transformation framework [23]. In their tool *MOTOE* [15], Kessentini et al. extract transformation blocks from examples and use Particle Swarm Optimisation (PSO) as a search technique. In general, approaches that use meta-heuristics can potentially scale better than exact search-based approaches, but have to sacrifice hard guarantees of correctness, completeness, and optimality of partial solutions.

## 5 Preliminary Definitions

Our basic definitions are adapted from Ehrig et al. [9], supplemented by the definition of schema compliance [3]. TGGs are a declarative rule-based approach which describes a language of triples of *graphs*. For that, we use the categorical definition of graphs, treating graphs as objects and *graph morphisms* as arrows, injectively mapping elements of one graph to those of another.

**Definition 1 (Graph (Morphism)).**
*A* ***graph*** $G = (V, E, src, trg)$ *consists of a set* $V$ *of nodes (vertices), a set* $E$ *of edges, and two functions* $src, trg : E \rightarrow V$ *that assign each edge a source and target node, respectively. The set* $elem(G) = V \cup E$ *denotes the union of vertices and edges. Given graphs* $G = (V, E, src, trg)$, $G' = (V', E', src', trg')$, *a* ***graph morphism*** $f : G \rightarrow G'$ *consists of two functions* $f_V : V \rightarrow V'$ *and* $f_E : E \rightarrow E'$ *such that* $src \,;f_V = f_E \,;src'$ *and* $trg \,;f_V = f_E \,;trg'$. *The* $;$ *operator denotes the composition of functions:* $f \,;g(x) := g(f(x))$.

Based on Def. 1 triple graphs and triple morphisms can also be defined categorically. A *triple graph* consists of a correspondence graph with a unique morphism to a source graph and a target graph each. An example for such a triple graph is depicted in Fig. 8. Source and target graph are interchangeable, such that the choice for source and target between the `Facebook` model and the `Instagram` model is just a question of design.

**Definition 2 (Triple Graph (Morphism)).**
*A* ***triple graph*** $G = G_S \xleftarrow{\gamma_S} G_C \xrightarrow{\gamma_T} G_T$ *consists of graphs* $G_S, G_C, G_T$ *and graph morphisms* $\gamma_S : G_C \rightarrow G_S$ *and* $\gamma_T : G_C \rightarrow G_T$. *elem(G) denotes the union elem(*$G_S$*)* $\cup$ *elem(*$G_C$*)* $\cup$ *elem(*$G_T$*). A* ***triple morphism*** $f : G \rightarrow G'$ *with* $G' = G'_S \xleftarrow{\gamma'_S} G'_C \xrightarrow{\gamma'_T} G'_T$, *is a triple* $f = (f_S, f_C, f_T)$ *of graph morphisms where* $f_X : G_X \rightarrow G'_X$, $X \in \{S, C, T\}$, $\gamma_S \,;f_S = f_C \,;\gamma'_S$ *and* $\gamma_T \,;f_T = f_C \,;\gamma'_T$.

In this setting, we introduce typing by demanding a type (triple) morphism to a chosen type (triple) graph. In Fig. 5, network nodes and user nodes can be distinguished by typing information, for instance. The language of a type (triple) graph $TG$ is the set of (triple) graphs typed over $TG$.

**Definition 3 (Typed Triple Graph (Morphism)).**
***A typed triple graph*** $(G, type)$ *is a triple graph* $G$ *together with a triple morphism* $type : G \rightarrow TG$ *to a distinguished type triple graph* $TG$. *A* ***typed triple***

***morphism*** *$f : \hat{G} \to \hat{G}'$ is a triple morphism $f : G \to G'$ with $type = f; type'$, where $\hat{G} = (G, type), \hat{G}' = (G', type')$. $\mathcal{L}(TG) := \{G \mid \exists\ type : type(G) = TG\}$ denotes the set of all triple graphs of type $TG$.*

In the following, all (triple) graphs and (triple) morphisms are assumed to be typed unless explicitly stated otherwise. A (triple) graph morphism can be viewed as a *monotonic (triple) rule*, such as depicted in Fig. 4, 5, 6 or 7 of the running example. By applying a (triple) rule on a concrete host graph, nodes and edges can be added to produce a new triple. (Triple) rules are applied by constructing a *pushout*, which can be interpreted as a generalised union of (triple) graphs $R$ and $G$ over a common sub-(triple)graph $L$:

**Definition 4 (Triple Rule (Application)).**
*A **triple rule** $r : L \to R$ is a monomorphic (injective) triple morphism. A direct derivation $G \overset{r@m}{\Longrightarrow} G'$ via a triple rule $r$, is constructed as depicted to the right by building a pushout over $r$ and a triple monomorphism $m : L \to G$ called a match. A **derivation** $D : G \overset{*}{\Longrightarrow} G_n = G \overset{r_1@m_1}{\Longrightarrow} G_1 \overset{r_2@m_2}{\Longrightarrow} \cdots \overset{r_n@m_n}{\Longrightarrow} G_n$ is a sequence of direct derivations. We denote by $\mathcal{D} = \{d_1, \ldots, d_n\}$ the underlying set of direct derivations included in $D$.*

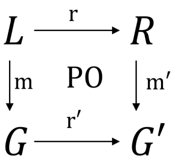

Starting off with the *empty triple graph*, all triples that can be produced by finitely many rule applications form the *language* of a TGG.

**Definition 5 (Triple Graph Grammar (Language)).**
*A **triple graph grammar** $TGG = (G, \mathcal{R})$ consists of a triple graph $G$, and a finite set $\mathcal{R}$ of triple rules. The **triple graph language** of $TGG$ is defined as $L(TGG) = \{G_\emptyset\} \cup \{G \mid \exists\ D : G_\emptyset \overset{*}{\Longrightarrow} G\}$, where $G_\emptyset$ is the **empty triple graph**.*

While the formal definition of rule-based triple graph generation is completed at this point, we want to pose further restrictions on triples by introducing domain constraints. Therefore, we introduce graph conditions for triple graphs and graph constraints as a context-independent form of graph conditions. A graph constraint is either satisfied trivially, if there does not exist a match for the premise $P$, or if there exists at least one match for a conclusion $C_i$.

**Definition 6 (Graph Constraint).**
*A **graph constraint** is a pair $gc = (p_\emptyset : G_\emptyset \to P, \{c_i : P \to C_i \mid i \in I\})$, for some index set $I$. $P$ is referred to as the **premise** and $\{C_i \mid i \in I\}$ as the **conclusions** of the graph constraint $gc$. A triple graph $G$ **satisfies** $gc$, denoted by $G \models gc$, iff $\forall\, m_p : P \to G, \exists\, i \in I\, \exists\, m_{c_i} : C_i \to G, [m_p = c_i; m_{c_i}]$, where $m_p, (m_{c_i})_{i \in I}$ are monomorphisms.*

A type graph $TG$ along with a set of graph constraints is denoted as *schema* for graphs. In the running example, the schema consist of the metamodel (Fig. 1) and the graph constraints depicted in Fig. 2 and 3. A (triple) graph *complies* to a schema if it is typed over $TG$ and fulfils all graph constraints.

**Definition 7 (Schema Compliance).**
*A schema is a pair* $(TG, \mathcal{GC})$ *of a type triple graph* $TG$ *and a set* $\mathcal{GC} \subseteq \mathcal{L}(TG)$ *of graph constraints. Let* $\mathcal{L}(TG, \mathcal{GC}) := \{G \in \mathcal{L}(TG) \mid \forall gc \in \mathcal{GC},\ G \models gc\}$ *denote the set of all* ***schema-compliant*** *triple graphs.*

Finally, a triple graph is denoted as *consistent* with respect to a schema and a TGG if it is schema-compliant and contained in the language of the TGG.

**Definition 8 (Consistency).**
*Given a triple graph grammar* $TGG$ *and a schema* $(TG, \mathcal{GC})$*, a triple graph* $G$ *is said to be* ***consistent*** *iff* $G \in \mathcal{L}(TGG) \cap \mathcal{L}(TG, \mathcal{GC})$.

## 6 Correctness and Completeness

We now formalise our approach to guarantee *correctness* and *completeness*, i.e., the consistency check succeeds if and only if the input model is consistent. As our approach extends seminal work by Leblebici et al. [20], [18] and Weidmann et al. [24] towards graph constraints, large parts of the formalisation originate from these sources in an adapted version. The novelty of this section is the integration of graph constraints into this formal framework (Def. 10, 12, 15, 18), as well as showing that formal properties still hold in a setting with graph constraints (Def. 21 ff.), assuming that the TGG at hand is progressive (Def. 23), i.e. each rule application marks at least one element.

In the original definition of TGGs (Def. 5), triples are *generated* by creating elements in source, correspondence and target graph simultaneously. For consistency checking, a TGG can be *operationalised* to check if a given triple is contained in the language of a TGG. In this case, elements are *marked* by rule applications instead of being created. To determine if a concrete triple graph is a member of the language of a TGG, one searches for a derivation sequence starting with the empty triple graph (cf. Def. 5) and producing the triple graph. The consistency checking operation derived from a TGG does not modify the input triple but instead marks this graph by successive rule applications in the course of a derivation sequence. An operational rule, derived from a corresponding triple rule, requires its context elements to be marked already.

**Definition 9 (Operational Rule and Marking Elements).**
*Given a triple rule* $r : L \to R$*, the* ***operational rule*** $cr : CL \to CR$ *for* $r$ *is constructed as depicted to the right. It holds* $CL = CR = R$*, and* $cr : CL \to CR = id_{CR}$*. An element* $e \in elem(R)$ *is a* ***marking element*** *of* $cr$ *iff* $\nexists e' \in elem(L)$ *with* $r_S(e') = e$ *or* $r_C(e') = e$ *or* $r_T(e') = e$.

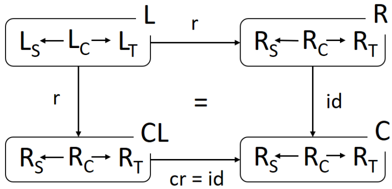

For operational rules, elements can be partitioned into those which are created by the original TGG rule (marked elements) and those which must be provided as context (required elements). Graph constraints do not mark elements and therefore, only a set for the elements required by premise and conclusion, respectively, are defined.

**Definition 10 (Marked and Required Elements).**
*For a direct derivation* $d : G \overset{cr@cm}{\Longrightarrow} G$ *via an operational rule* $cr : CL \to CR$*, the following sets are defined:*

- *mrk(d)* $= \{e \in$ *elem(G)* $\mid \exists\, e' \in$ *elem(CL)*, $cm(e') = e$ *where* $e'$ *is a marking element of cr*$\}$
- *req(d)* $= \{e \in$ *elem(G)* $\mid \exists\, e' \in$ *elem(CL)*, $cm(e') = e$ *where* $e'$ *is not a marking element of cr*$\}$

*For a graph constraint* $gc = (p_\emptyset : G_\emptyset \to P, \{c_i : P \to C_i \mid i \in I\})$*, we define:*

- $req(p_\emptyset) = \{e \in elem(G) \mid e' \in elem(P), m_p(e') = e\}$
- $req(c_i) = \{e \in elem(G) \mid e' \in elem(C_i), m_{c_i}(e') = e\}, i \in I$

All candidate rule applications are associated with a binary variable which indicates by its value (0 or 1) whether the candidate is considered within the final solution. To determine the variable assignment, all candidates are collected and handed over to an ILP solver to determine the optimal subset of rule applications (cf. Sect. 2) respecting all linear constraints.

**Definition 11 (Constraints for Derivations).**
*Given a triple graph* $G$*, let* $D : G \overset{*}{\Longrightarrow} G$ *be a derivation via operational rules with the underlying set* $\mathcal{D}$ *of direct derivations. For each direct derivation* $d_1, \ldots, d_n \in \mathcal{D}$*, respective binary variables* $\delta_1, \ldots, \delta_n$ *with* $\delta_1, \ldots, \delta_n \in \{0, 1\}$ *are defined. A linear constraint* $\mathcal{LC}$ *for* $\mathcal{D}$ *is a conjunction of linear inequalities which involve* $\delta_1, \ldots, \delta_n$*. A set* $\mathcal{D}' \subset \mathcal{D}$ *fulfils* $\mathcal{LC}$*, denoted as* $\mathcal{D}' \vdash \mathcal{LC}$*, iff* $\mathcal{LC}$ *is satisfied for variable assignments* $\delta_i = 1$ *if* $d_i \in \mathcal{D}'$ *and* $\delta_i = 0$ *if* $d_i \notin \mathcal{D}', 1 \le i \le n$*.*

Graph constraints are also associated to binary variables to ensure that only schema-compliant triples pass the consistency check, while premises and each of the corresponding conclusions are split into separate constraints. In contrast to the binary variables for rule applications, the value assignment cannot be chosen by the ILP solver. Instead, any variable assignment which does not violate the linear constraints is fine, as they ensure schema-compliance by the interrelations of rule applications and graph constraints.

**Definition 12 (Constraints for Graph Constraints).**
*Let* $\mathcal{GC} = \{(p_\emptyset : G_\emptyset \to P, \{c_i : P \to C_i \mid i \in I\})\}$ *be a set of graph constraints. For each graph constraint* $gc \in \mathcal{GC}$*, respective binary variables* $\pi_1 \ldots \pi_n$ *for the premises and* $\gamma_{1,1} \ldots \gamma_{1,m_1} \ldots \gamma_{n,1} \ldots \gamma_{n,m_n}$ *for the conclusions are defined. A linear constraint* $\mathcal{LC}$ *for* $\mathcal{GC}$ *is a conjunction of linear inequalities which*

*involve* $\pi_1 \dots \pi_n$ *and* $\gamma_{1,1} \dots \gamma_{1,m_1} \dots \gamma_{n,1} \dots \gamma_{n,m_n}$. *A triple graph G fulfils* $\mathcal{LC}$, *denoted as* $G \models \mathcal{LC}$, *iff* $\mathcal{LC}$ *is satisfied for any variable assignment* $\{\pi_1 \dots \pi_n\} \to \{0,1\}, \{\gamma_{1,1} \dots \gamma_{1,m_1} \dots \gamma_{n,1} \dots \gamma_{n,m_n}\} \to \{0,1\}$.

As the operational rules reflect the behaviour of the original rules of the underlying TGG, multiple markings for the same elements must be prohibited as this would mean that an element is created multiple times. For each node and edge, a linear constraint is created that ensures that this element is marked at most once in order to guarantee schema compliance and containment in the language of the TGG later on.

**Definition 13 (Sum of Alternative Markings for an Element).** *Given a triple graph G, let* $D : G \overset{*}{\Longrightarrow} G$ *be a derivation via operational rules with the underlying set* $\mathcal{D}$ *of direct derivations. For each element* $e \in elem(G)$, *let* $\mathcal{E}(e) = \{d \in \mathcal{D} \mid e \in mrk(d)\}$. *The integer mrkSum(e) denotes the sum of the associated variable assignments for each* $d \in \mathcal{E}$:

$$mrkSum(e) = \sum_{d_i \in \mathcal{E}(e)} \delta_i$$

**Definition 14 (Constraint 1: Mark Elements at Most Once).** *Given a triple graph G, let* $D : G \overset{*}{\Longrightarrow} G$ *be a derivation via operational rules:*

$$markedAtMostOnce(G) = \bigwedge_{e \in elem(G)} [\ mrkSum(e) \leq 1]$$

The reason for the sum of marked elements not being strictly equal to 1 is the desired treatment of inconsistent inputs: The system should still be feasible in case of inconsistent inputs and a maximal consistent sub-triple should be the result of the optimisation step.

The following constraint ensures that the required context elements for operational rule applications as well as premises and conclusions are provided in the final solution, such that the original TGG rule is guaranteed to be applicable in this situation and the marked part of the triple graph is schema-compliant.

**Definition 15 (Constraint 2: Guarantee Context).** *Given a triple graph G and a schema* $(TG, \mathcal{GC})$, *let* $D : G \overset{*}{\Longrightarrow} G$ *be a derivation via operational rules with the underlying set* $\mathcal{D}$ *of direct derivations. For each direct derivation* $d \in \mathcal{D}$ *and each graph constraint* $gc \in \mathcal{GC}$, *the following constraints are defined:*

$$con(d) = \bigwedge_{e \in req(d)} [\delta \leq mrkSum(e)]$$

$$con(p_\emptyset) = \bigvee_{e \in req(p_\emptyset)} [\ mrkSum(e) \leq \pi]$$

$$con(c_i) = \bigwedge_{e \in req(c_i)} [\gamma_i \leq mrkSum(e)], i \in I$$

$$context(D) = \bigwedge_{d \in \mathcal{D}} con(d) \wedge \bigwedge_{gc \in \mathcal{GC}} [con(p_\emptyset) \wedge \bigwedge_{i \in I} con(c_i)]$$

There are constellations in which rule application candidates mutually provide context for each other in a *dependency cycle*, such that parts of the graph could be potentially marked by these rules, but none of them can ever be applied first because the necessary context is not yet there. Therefore, we introduce a relation $\rhd$ among rule applications to arrange them in a proper order.

**Definition 16 (Dependency Cycles).**
*Let $D : G \stackrel{*}{\Longrightarrow} G$ be a derivation via operational rules with the underlying set $\mathcal{D}$ of direct derivations. A relation $\rhd \subseteq \mathcal{D} \times \mathcal{D}$ between $d_i, d_j \in \mathcal{D}$ is defined as follows:*

$$d_i \rhd d_j \text{ iff } req(d_i) \cap mrk(d_j) \neq \emptyset$$

*A set $cy \subseteq \mathcal{D}$ with $cy = \{d_1, \ldots, d_n\}$ of direct derivations is a* ***dependency cycle*** *iff $d_1 \rhd \cdots \rhd d_n \rhd d_1$.*

The following constraint breaks dependency cycles by forbidding to choose all of its member rule applications for the final solution.

**Definition 17 (Constraint 3: Forbid Dependency Cycles).**
*Given a triple graph $G$, let $D : G \stackrel{*}{\Longrightarrow} G$ be a derivation via operational rules with the underlying set $\mathcal{D}$ of direct derivations, and let $\mathcal{CY}$ be the set of all dependency cycles $cy \in \mathcal{D}$. A linear constraint acyclic(D) is defined as follows:*

$$acyclic(D) = \bigwedge_{cy \in \mathcal{CY}, cy = \{d_1, \ldots, d_n\}} \sum_{i=1}^{n} \delta_i < n$$

While the previous constraint types guarantee containment in the language of the TGG at hand as well as context constraints for premises and conclusions, Constraint 4 expresses the semantics of graph constraints to achieve schema-compliance. Thereby, the linear constraint is very similar to the definition for satisfaction of graph constraints (Def. 6). It is possible to formulate this constraint independent of the concrete rule application because only graph constraints are supported instead of arbitrary graph conditions.

**Definition 18 (Constraint 4: Satisfy Graph Constraints).**
*Let $(TG, \mathcal{C} = \{(p_\emptyset : G_\emptyset \to P, \{c_i : P \to C_i \mid i \in I\})\})$ be a schema. A linear constraint $sat(G)$ expressing that $G$ fulfils all graph constraints of $\mathcal{C}$ is defined as follows:*

$$sat(G) = \bigwedge_{C \in \mathcal{C}} [\neg\pi \vee \bigvee_{i \in I} \gamma_i]$$

Finally, the objective function can be defined to maximize the number of markings over the entire input triple, while ensuring that no correctness constraints are violated and the result is schema-compliant according to Def. 7.

**Definition 19 (Optimisation Problem).**
*Given a triple graph G and a schema (TG,C), let* $D : G \overset{*}{\Longrightarrow} G$ *be a derivation via operational rules. The ILP to be optimised is constructed as follows: max.* $\sum_{d \in D} |mrk(d)|$ *s.t. markedAtMostOnce(G)* $\wedge$ *context(D)* $\wedge$ *acyclic(D)* $\wedge$ *sat(G)*

The remainder of this section provides a proof sketch showing that the consistency check always terminates, and succeeds iff the input triple graph is consistent with respect to Def. 8. It is an extension of the proof for correctness and completeness in a setting without graph constraints [18,24], such that the focus of this version is set on schema compliance. In the following, let a TGG $TGG = (G_\emptyset, \mathcal{R})$, a schema $(TG, \mathcal{GC})$, a triple graph $G$, and a derivation via operational rules $D : G \overset{*}{\Longrightarrow} G$ with underlying set of direct derivations $\mathcal{D}$ be given for all definitions, lemmas and theorems.

First, we define a *proper subset* of operational rule applications as a set which is associated to a feasible solution for the ILP (Def. 14, 15, 17 and 18).

**Definition 20 (Proper Subset of Rule Applications).**
*A subset* $\mathcal{D}' \subseteq \mathcal{D}$ *is a proper subset of* $\mathcal{D}$ *iff* $\mathcal{D}' \vdash$ *markedAtMostOnce*$(G) \wedge$ *context*$(D) \wedge$ *acyclic*$(D) \wedge$ *sat*$(G)$.

Next, it is shown that there exists a sequence of the rule applications of a proper subset, such that the marked elements of the graph form a consistent triple. Furthermore, the marked part of the graph is schema-compliant.

**Lemma 1 (Consistent Portions of a Triple Graph).**
$\exists$ *proper subset* $\mathcal{D}' \subseteq \mathcal{D} \iff \exists G' \in L(TGG) \cap \mathcal{L}(TG, \mathcal{GC})$ *such that:*

$$elem(G') = \bigcup_{d' \in \mathcal{D}'} mrk(d')$$

*Proof (Sketch).* When all direct derivations $d \in \mathcal{D}'$ are sequenced over the $\triangleright$ relation (Def. 16), a proper subset according to Def. 20 is formed, resulting in a triple graph $G' \in L(TGG)$ consisting of the elements marked by $D'$. At the same time, $G'$ will be schema-compliant iff $\mathcal{D}' \vdash \text{sat}(G')$ as this predicate ensures that all given graph constraints are satisfied.

We demand the property of *maximality* to avoid trivial solutions such as the empty triple graph:

**Definition 21 (Maximal Proper Subset of Rule Applications).**
*A proper subset* $\mathcal{D}'$ *of* $\mathcal{D}$ *is* maximal *if there does not exist any other proper subset* $\mathcal{D}''$ *of* $\mathcal{D}$ *with a greater objective function value (cf. Def. 19).*

The application of a sequenced maximal proper subset of rule applications on the empty triple graph is denoted as *maximally marked triple graph.*

**Definition 22 (Maximally Marked Triple Graph).**
*Let* $\mathcal{D}'$ *be a maximal, proper subset of* $\mathcal{D}$*. The triple graph* $G'$ *identified with* $\mathcal{D}'$ *according to Lemma 1 is denoted as a* maximally marked triple graph *with respect to D.*

Theorem 1 guarantees that a triple graph that can be completely marked by rule applications of a maximal proper subset is indeed consistent.

**Theorem 1 (Correctness).**
*For a maximally marked triple graph $G'$ with respect to $D$, it holds:*

$$\bigcup_{d \in \mathcal{D}} mrk(d) = elem(G) \implies G' \text{ is consistent}$$

*Proof (Sketch).* $G' \in L(TGG)$ immediately follows from Lemma 1: As $D$ is a maximal proper subset, $G' \in L(TGG)$ holds, and the rule applications of $D$ can be sequenced, such that they can mark $G'$ entirely according to the premise of this theorem. $G' \in L(TG, \mathcal{GC})$ holds as well because the choice of any $d \in D'$ leading to a violation of any $gc \in \mathcal{GC}$ would make $sat(G')$ false. Therefore, $G'$ is consistent according to Def. 8.

To guarantee completeness, it remains to show that the process of constructing the ILP terminates, which requires the set of possible rule applications to be finite. As all possible derivation sequences are collected, the ILP solver terminates with an optimum solution iff one exist. We therefore demand the underlying TGGs to be *progressive*, i.e., each operational rule is required to mark at least one element. In fact, operational rules that do not mark elements correspond to TGG rules that do not have any effect on the host graph they are applied on because they cannot add any elements, and are therefore irrelevant for practical use.

**Definition 23 (Progressive TGGs).**
*TGG is progressive if each of its operational rules has at least one marking element.*

Demanding the TGG at hand to be progressive, completeness can be concluded by showing that the consistency check cannot cycle.

**Theorem 2 (Completeness).**
*Let TGG be progressive. A maximally marked triple graph $G'$ with respect to $D$ exist such that:*

$$G' \text{ is consistent} \implies \bigcup_{d \in \mathcal{D}} mrk(d) = elem(G)$$

*Proof (Sketch).* As Lemma 1 guarantees the existence of a derivation $D$, and ILP solving always produces a maximally marked triple graph $G'$, we only need to show the implication (equivalence follows from Thm. 1). To derive a contradiction, we now assume that $G'$ is consistent, but that $G'$ either contains unmarked elements or violates any constraint $gc \in \mathcal{GC}$. From $G'$ being consistent, it follows from the decomposition and composition theorem for TGGs and operational rules [8, 18] that there exists a derivation sequence $D' : G \overset{*}{\Longrightarrow} G'$ with operational rules. This means that at least one rule application of $D'$ is not contained in $D$ or $G'$ violates any $gc \in \mathcal{GC}$. The latter is impossible, as it would contradict to the assumption that $G'$ is consistent. The former implies that the objective function value could be increased by using $D'$ for marking $G$, which contradicts the optimality of the result found by ILP solving.

## 7 Implementation and Experimental Evaluation

We investigate the impact of graph constraints on runtime performance, considering scalability of consistency checking for growing model sizes with and without taking graph constraints into account, by two research questions:

(RQ1) By which factor does the number of variables and ILP constraints increase when introducing graph constraints to the ILP? How does this influence the runtime of pattern matching, ILP construction, and ILP solving?
(RQ2) How does the runtime performance relate to model size (number of nodes and edges) for consistency checking with and without graph constraints?

**Setup:** We implemented our approach within the tool eMoflon[2] using Neo4J[3] as an underlying graph pattern matcher and database for querying and storing the models. As a test example, we took the `FacebooktoInstagram` TGG as described in Sect. 2. To obtain synthetic models, we used the derived TGG-based model generator to produce random models with 1078 to 226,988 elements (roughly the same number of nodes and edges). We then executed the derived TGG-based consistency checker, once taking the negative graph constraint from Sect. 2 into account, and once without any graph constraints. For each configuration, the number of variables and constraints of the ILP, as well as the time needed for pattern matching, ILP construction, and ILP solving were measured for 10 repeated runs. As final values, the medians of the 10 test runs were taken to minimize the bias introduced by outliers. All performance tests were executed on a standard notebook with an Intel Core i7 (1.80 GHz), 16GB RAM, and Windows 10 64-bit as operating system. An installation of Eclipse IDE for Java and DSL Developers, version 2019-09 with Java Development Kit (JDK) version 13 was used. The JVM running the tests was allocated a maximum of 4GB memory, and 8GB were allocated to the graph database Neo4J.

**Results:**[4] Figure 9 shows the time needed for pattern matching, ILP construction, and ILP solving for different model sizes. One can observe that for both configurations (with and without graph constraints), the runtime of all components depends linearly on the number of model elements. Taking graph constraints into account for the consistency check makes the ILP construction roughly 20% - 40% slower. This is to be expected as the ILP problem is simply larger. For similar reasons, a difference can also be observed for the ILP solving step, whose runtime is negligible without constraints, but increases by a factor of 10 when including graph constraints. While this increase is substantial, ILP solving does not have a large overall impact on the runtime performance even for 200k elements. Interestingly, pattern matching gets faster when the additional negative graph constraint is included. This is surprising as additional pattern matching is required to determine matches of the negative constraint. The underlying graph database is heuristic-based, however, and also uses caching strategies to decide what data to keep in memory. Apparently the pattern matching strategy applied for the collection of patterns including the negative constraint seems to scale better for model sizes greater than 130k.

[2] `github.com/eMoflon/emoflon-neo` [3] `neo4j.com` [4] `bit.ly/2BFAutd`

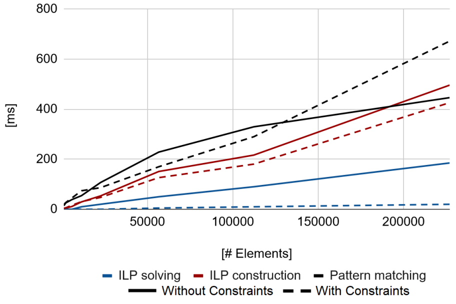

**Fig. 9.** Runtime Measurements

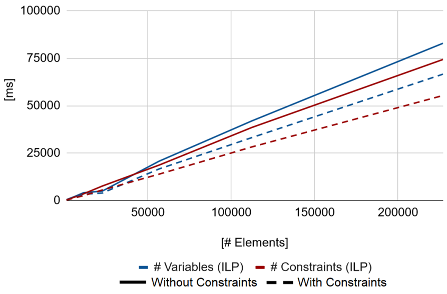

**Fig. 10.** #Variables and #Constraints

The number of binary variables and constraints grows linearly with model size for both settings, involving slightly more variables than constraints (cf. Fig. 10). With the negative graph constraint, this number increases by about 25%-50%.

**Summary:** Revisiting our research questions, one can state that the number of binary variables and constraints increases by a constant factor when introducing (negative) graph constraints, resulting in a constant increase of the overall runtime for consistency checking. While the ILP solving step increases substantially and could become problematic for large models, our measurements indicate that the ILP solving step is probably not the bottle neck for our example (RQ1). In both settings (with and without the negative graph constraint), the runtime for consistency checking increases linearly with growing model size (RQ2).

**Threats to validity:** The evaluation was performed with only one TGG consisting of only four rules, only the consistency checker (of all operations) was run on randomly generated synthetic instances, and we measured the additional price of taking only the negative graph constraint from Sect. 2 into account. While our initial results are positive and indicate that the additional price of guaranteeing schema compliance as we propose does not render the ILP-based TGG operations infeasible due to an explosion in runtime, extensive benchmarking with multiple TGGs, multiple graph constraints, larger model sizes, and multiple consistency management operations is required to transfer these results to practical, real-world applications.

## 8 Extension to Other Operations

The presented concepts are tailored to consistency checking with correspondences, i.e. source, target and correspondence model are given as inputs and are *marked* by operational rule applications, whereas all three models are simultaneously *created* by the original rule applications. There are also other operations which use a mixture of creating and marking elements to complement given input models to a complete triple. Figure 11 depicts the example instance of Fig. 8 annotated with the operations which require the respective model(s) as input. The previously presented CO (check only) operation gets all three models as input, whereas CC (correspondence creation) checks for consistency by building up the correspondence model for given source and target models. FWD_OPT and BWD_OPT are operations for unidirectional transformation, i.e. either the source or the target model is given and a consistent transformation to the respective other domain is computed. A formal specification of the operations was introduced by Weidmann et al. [24].

All these operations are based on a common formalism that expresses dependencies between rule applications as ILP constraints, while in contrast to the definitions of this paper, dependencies between created elements are also taken into account. As constraints for marked and created parts of the triple are formed almost the same way, it is possible to transfer the results for consistency checking respecting graph constraints to the other operations as well. However, the formal proof which guarantees the operations' correctness and completeness [18,24] has to be extended to take graph constraints into account.

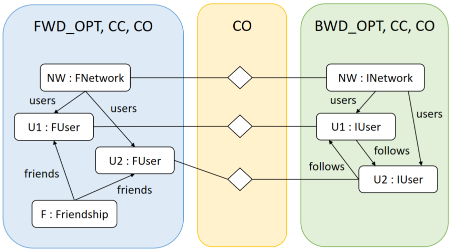

**Fig. 11.** Input models per operation

## 9 Conclusion and Future Work

We presented an extension of a seminal approach to combining TGGs and ILP by supporting graph constraints. For consistency checking with given correspondence links, we have shown correctness and completeness of the approach. The results can be generalised towards other operations such as unidirectional transformations as well. Additionally, the approach was implemented in a TGG tool, and an experimental evaluation indicated that the scalability of the approach is sufficient for practical use. For future work, we plan to extend the approach to cope with general AC as well, increasing the expressive power of the supported class of TGGs. As a proof of concept, we only implemented negative constraints until now, which should be extended towards general graph constraints. Using an incremental pattern matcher with extensible matches, it should be possible to collect matches for the premise and corresponding conclusions at once, which would keep the implementation efficient. Further performance tests with other (industrial) examples will also be necessary to underpin the validity of the evaluation results with respect to runtime performance, as both the metamodels and the rule set are very restricted, whereas the considered model sizes are realistic. Generating consistent models first and then mutate them slightly would further lead to a smaller and therefore more reasonable number of inconsistencies.

## Acknowledgements

We like to thank Surbhi Verma, Shubhangi Salunkhe and Darya Zarkalam for contributing to large parts of the implementation.
